# Arrhythmic Risk and Treatment after Transcatheter Atrial Septal Defect Closure

**DOI:** 10.3390/diagnostics14010033

**Published:** 2023-12-23

**Authors:** Silvia Deaconu, Alexandru Deaconu, Gabriela Marascu, Mihaela Octavia Stanculescu, Dragoș Cozma, Eliza Cinteza, Radu Vatasescu

**Affiliations:** 1 ARES Centers, 021967 Bucharest, Romania; si.deaconu@gmail.com; 2 Cardiology Department, Clinic Emergency Hospital, 014461 Bucharest, Romania; gabriela.marascu@yahoo.com (G.M.); mihaela.octavia@yahoo.com (M.O.S.); radu.vatasescu@umfcd.ro (R.V.); 3 Cardio-Thoracic Department, “Carol Davila” University of Medicine and Pharmacy, 050474 Bucharest, Romania; 4 Department of Cardiology, “Victor Babes” University of Medicine and Pharmacy, 2 Eftimie Murgu Sq., 300041 Timisoara, Romania; dragoscozma@gmail.com; 5 Department of Pediatrics, “Carol Davila” University of Medicine and Pharmacy, 050474 Bucharest, Romania; elizacinteza@yahoo.com; 6 “Marie Skolodowska Curie” Emergency Children’s Hospital, 041451 Bucharest, Romania

**Keywords:** atrial septal defect, atrial arrhythmias, sudden cardiac death

## Abstract

Atrial septal defect (ASD) represents the most common congenital heart defect identified in adulthood. Atrial and ventricular geometric remodeling due to intracardiac shunt increase the risk of arrhythmias, especially atrial fibrillation (AF). Clinical, echocardiography, electrocardiogram, and device-related predictors may be used to assess the risk of atrial arrhythmias after ASD closure. The underlying mechanisms in these patients are complex and at least in part independent of the structural remodeling secondary to hemodynamic overload. Device closure of the ASD itself and its timing impact future arrhythmia risk, as well as posing a challenge for when transseptal puncture is required. Sudden cardiac death (SCD) risk is higher than in the general population and an implantable cardioverter-defibrillator (ICD) may be indicated in selected cases.

## 1. Introduction

Among congenital heart diseases, atrial septal defect (ASD) has always held researchers’ and clinicians’ attention. Although seemingly straightforward, ASD causes a cascade of physiological consequences as it results in an abnormal opening in the septum between the heart’s atria. ASD is one of the most common congenital heart diseases (CHDs) and in its natural history can be associated with the development of arrhythmias, right heart failure, stroke, and pulmonary hypertension [1]. However, the main cause of morbidity and mortality in ASD patients is attributed to the development of atrial tachyarrhythmias [2]. Among several types of ASD, ostium secundum ASD may be closed surgically with open heart surgery or interventionally by using a percutaneous atrial septal occluder (ASO). The effects of ASD interventional closure on the prevalence and evolution of atrial arrhythmias are controversial. Moreover, the presence of the ASO limits access to the left atrium. 

Developing insights into cardiac pathology has revealed a compelling connection between ASD and cardiac arrhythmia risk, challenging traditional perspectives and necessitating a nuanced exploration. By examining the mechanisms linking ASD to cardiac arrhythmias, we aim to provide a synthesis of current knowledge, providing insight into diagnostic challenges, prognostic factors, and therapeutic interventions. Specifically, we will focus on establishing arrhythmia types and their predictors related to the presence of an ostium secundum ASD and management after the implantation of an ASD closure device. 

## 2. Arrhythmias and Conduction Disorders Associated with ASD

Patients with ASD and a shunt ratio ≥ 2.5 are at high risk for arrhythmias [3]. Volume overload secondary to left-to-right atrial shunt leads to atrial structural remodeling, which is more pronounced in the right atrium and frequently results in supraventricular arrhythmias, especially atrial flutter and atrial fibrillation (AF) [4]. Several studies showed fibrosis and electrophysiological anomalies in both atria, including reduced voltages, prolonged atrial refractory periods, spatial heterogeneity of conduction, and abnormalities in the Bachmann interatrial bundle [4,5].

Right ventricular (RV) volume and pressure overload leads to RV geometrical and electrical remodeling, with a shifted interventricular septum toward the left ventricle, leading to impaired diastolic filling, reduced stroke volume, and possibly fibrosis over time [4]. However, although current cardiac magnetic resonance imaging (cMRI) data convincingly demonstrated RV dilation, they were not able to provide evidence for significant RV fibrosis even in adult ASD patients [6,7,8].

The risk of ventricular arrhythmias in ASD is not conclusively proved, although there are reported cases of sudden cardiac death (SCD) in both repaired and unrepaired ASD [9,10]. A retrospective study that included 1000 patients with transcatheter ASD closure showed an increased risk for ventricular disturbances and atrioventricular conduction disorders [11]. Udholm et al. found that non-sustained ventricular tachyarrhythmias (NSVTs) were present in 8% patients with small ASD and suggested a potential and irreversible progression in the hemodynamic significance of ASD [12]. On the other hand, Koyak et al. showed that NSVT was not associated with SCD in patients with CHD [10]. 

In addition to atrial fibrillation and cavo-tricuspid isthmus (CTI) atrial flutter, other arrhythmias and conduction disorders can develop after percutaneous ASD closure like focal or other re-entrant atrial tachycardias, sinus node dysfunction, and complete heart block. Although atrioventricular reentry tachycardia and non-sustained supraventricular tachycardia are not more common than in the general population, they may have additional challenges in ASD patients when submitted for catheter ablation [13].

A study conducted by Clark which included 15 patients with secundum ASD showed that 5 patients presented sinus node dysfunction or atrioventricular node dysfunction before repair [14]. On the other hand, atrioventricular block post-transcatheter closure of ASD occurs in rare instances, and it can be transient or may progress to complete heart block [15]. When compared with patients with unclosed ASD, Albaek et al. showed that those with interventional ASD closure were at higher risk of developing atrioventricular block, incomplete right bundle branch block, or left anterior fascicular block and had a higher demand for pacemaker implantation [16].

Other patients can develop persistent arrhythmias due to the closure device itself, explained by the formation of a device-related reentrant circuit, anisotropic conduction due to inflammation and scarring around the device, or Bachmann’s bundle deterioration [9,17]. 

**Figure 1:**
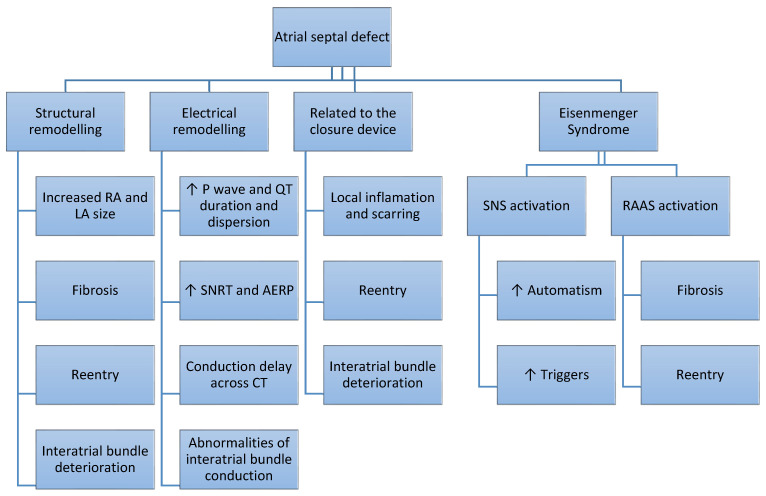
Arrhythmogenesis in patients with atrial septal defect. RA—right atrium; LA—left atrium; SNRT—sinus node recovery time; AERP—atrial effective refractory period; CT—crista terminalis; SNS—sympathetic nervous system; RAAS—renin angiotensin aldosterone system.

The main mechanisms involved in the arrhythmogenesis of patients with ASD are summarized in Figure 1.

### 2.1. Patients with Early-Onset Arrhythmias after ASD Closure

This category includes patients who experience transient arrhythmias days to weeks after ASD closure and resolve within one year post procedure [9]. Komar et al. showed that supraventricular premature beats can occur in early follow-up, along with a small risk of atrioventricular conduction abnormalities and paroxysmal AF [17]. 

These arrhythmias are caused by local irritation and are not attributed to the extension of atrial remodeling or intracardiac shunt [9]. There does not seem to be any correlation between the type and size of the occluder and the occurrence of atrial arrhythmias after defect closure [18]. The device may trigger an inflammatory response within the atrial myocardial tissue due to a foreign body reaction, which could increase the likelihood of arrhythmias [18]. Also, patients with PFO show a close temporal proximity between AF and occluder placement [9,18]. After the procedure, early-onset arrhythmias in pediatric patients may be related to changes in hemodynamics due to clinical circumstances [13].

### 2.2. Patients with Late-Onset Arrhythmias after ASD Closure

This group includes patients who developed persistent arrhythmias. The incidence of long-term post-procedural arrhythmias is likely related to the progression of pre-existing atrial substrate abnormalities, increasing with age and the severity of remodeling markers secondary to ASD [9,19]. Geometrical remodeling is mostly observed on the right side of the heart, which experiences significant flow and pressure changes. However, individuals with ASD also exhibit geometrical changes on the left side, as evidenced by moderately increased LA dimensions on cardiac magnetic resonance and electroanatomical mapping [9]. These changes are also linked to electrical remodeling [9,19]. 

After the closure of ASD, older patients and those with higher pulmonary arterial pressure and larger right-sided chambers experience reduced cardiac geometric reverse remodeling. This puts them at a higher risk of developing arrhythmias, especially atrial fibrillation and atrial flutter [9]. It is also important to note that electrical reverse remodeling after ASD closure is limited compared to geometrical reverse remodeling, which can create and maintain an arrhythmogenic substrate [9]. 

On the other hand, the closure device may act as a central barrier of non-conduction, leading to the formation of a reentrant circuit around itself. Another hypothesis suggests that device closure may compromise interatrial conduction via Bachmann’s bundle, increasing arrhythmogenesis risk [9,18].

## 3. Predictors of Arrhythmias after ASD Interventional Closure

Percutaneous ASD closure is the gold standard treatment for the majority of cases, but its effects on the prevalence of atrial arrhythmias are still unclear. 

The main risk factors associated with the development of arrhythmias in patients with percutaneous ASD closure are presented in Table 1.

Timely interventional closure of ASD can reduce the incidence of arrhythmias due to the improvement in volume overload, and structural and electrical reverse remodeling in both atria [9,20]. However, long-term patients with interventional ASD closure present a higher risk of developing arrhythmias than the general population [9]. A meta-analysis of 25 studies demonstrated that transcatheter ASD closure is not associated with a reduction in the prevalence of any atrial arrhythmias. It showed a small benefit in patients ≥ 40 years but not those ≥ 60 years [21]. These findings suggest that the mechanisms underlying atrial arrhythmias in these patients are not fully understood and go beyond the ASD itself. Consequently, percutaneous closure alone may not be sufficient to treat atrial arrhythmias in many patients with ASDs, especially if not carried out in a timely manner. In patients who undergo ASD closure aged > 40 years, the prevalence of atrial arrhythmias is up to 40–60% [33]. If they develop symptomatic AF, these patients are managed similarly to those without congenital heart disease, with pulmonary vein isolation remaining the lesion set of choice, albeit access to the LA may be restricted after device closure [33].

Another meta-analysis conducted by Vecht et al. documented that surgical or percutaneous ASD closure reduced the prevalence of atrial arrhythmias in the short to mid-term but not at longer follow-up. Moreover, when only atrial fibrillation was considered, this effect was not seen in patients with ASD interventional closure [34]. One study suggested that in patients with AF, catheter ablation before ASD closure may be a feasible strategy for preventing AF recurrence [35].

The risk of long-term post-procedural arrhythmias increases with age and with the severity of remodeling and hemodynamic complications secondary to ASD [9]. 

Patients with ASD repaired over the age of 40 years can more often develop AF, explained by the dilatation of the pulmonary veins due to increased blood flow for long periods in the presence of a left-to-right shunt [20]. A prospective study that included 200 adults with transcatheter ASD closure showed that patients without a history of atrial tachyarrhythmias and those ˂ 40 years fared better, having the highest likelihood to be arrhythmia-free at a mean follow-up of 1.9 years [36]. A retrospective analysis showed that in patients > 40 years, the risk of developing new AF is about 6% during a 3-year follow-up [22]. Furthermore, at least 50% of the patients with paroxysmal atrial arrhythmia continue to have significant atrial arrhythmia following device closure [22]. 

Patients with patent foramen ovale who underwent percutaneous device repair can develop transient arrhythmias in the days or weeks post-closure, frequently due to local irritation [9]. Another study found that the risk of supraventricular arrhythmias in the first month after interventional ASD closure is increased by a larger device size and longer fluoroscopy time [17]. Jin et al. showed that patients with a ratio of atrial septal occluder versus atrial septal length > 0.576 might have a higher risk of arrhythmia during and after the closure procedures [32].

In a retrospective study which included 517 patients, Chiu et al. demonstrated that age, the size of the occluder—corresponding to the size of the ASD—fenestrated occluder, presence of multiple ASDs, pre-implantation atrial arrhythmia, metabolic syndrome, thyroid dysfunction, and mitral valve disease were significant risk factors for late atrial fibrillation or atrial flutter development [24]. Miura et al. showed that brain natriuretic peptide levels over 40 pg/mL before transcatheter ASD closure were associated with a high risk of new-onset atrial tachyarrhythmias after age adjustment [19].

Several studies concluded that QT or QTc duration and dispersion, T wave peak to end (Tp-e) interval, and Tp-e/QT ratio might be electrocardiographic susceptibility indexes for ventricular arrhythmias in ASD patients [29,31]. Also, increased P-wave duration post device closure can suggest an interatrial compromised conduction via Bachmann’s bundle [9]. Celik et al. showed that QRS fragmentation in inferior leads, called Crochetage sign, may predict incomplete right heart reverse remodeling and that it is significantly associated with late atrial arrhythmias [30].

Mansour et al. demonstrated that age, P wave dispersion, the systolic myocardial velocity of the right ventricle (S’), and systolic pulmonary artery pressure are independent risk factors for atrial arrhythmias in multivariate analysis [15]. Moreover, Van de Bruaene et al. proposed a risk score, in which male gender, atrial arrhythmia before repair, atrial arrhythmia ≤ 1 month, and systolic pulmonary artery pressure ≥ 25 mmHg are predisposing factors in the development of late atrial arrhythmias [23].

A study using CMR found that after transcatheter ASD closure, the LV remodels faster than the RV, while the RA shows reverse remodeling for up to 3 months. The RV remains enlarged 1 year later, and the LA volume does not change [37]. Left atrial dysfunction can be a predictor of the incidence and recurrence of atrial fibrillation in patients with successful transcatheter ASD closure and a history of atrial fibrillation [26]. On the other hand, Cakal et al. concluded that left atrial and left ventricular peak longitudinal strain and strain rate are not significantly affected in patients with ASD. Also, those parameters improved early after transcatheter correction but decreased 1 month post-procedure [38]. 

A prospective trial that included 73 patients who underwent percutaneous ASD closure showed that pre-existent right atrial dilatation and asynchrony assessed by 2D and 3D speckle tracking echocardiography had a significant association with paroxysmal atrial fibrillation independently of left atrial dysfunction [27].

Table 2 presents a comparative analysis of representative studies that evaluated the prevalence of atrial arrhythmias in patients with interventional ASD closure.

## 4. Long-Term Survival and Risk of SCD in Patients with ASD

Several studies demonstrated that mortality is higher for ASD patients than for the general population, and Eisenmenger syndrome, age at diagnosis, and pulmonary hypertension (PH) are independent positive predictors of mortality, while ASD closure is a positive independent predictor of survival [54,55]. SCD is a leading cause of death among adults with CHD, and accounts for up to 25% of deaths [56,57,58]. Various risk factors, such as conduction disturbances and ventricular dysfunction, have been previously identified as contributors to SCD [57]. The progression of QRS duration and the rate of deterioration of ventricular function can help to identify those at a higher risk of SCD [57].

Koyak et al. showed that an increase in QRS duration was often due to non-specific intraventricular conduction delay and predominantly seen in patients with Eisenmenger syndrome. The risk of SCD was reduced by 30% and 80% when QRS duration decreased by at least 5 ms/year and 10 ms/year, respectively [57]. Moreover, Moore et al. investigated 2935 patients with CHD and concluded that Eisenmenger syndrome had the highest risk category, resulting in 4.8 deaths per 1000 person-years incidence. A total of 338 ASD patients were included and two SCDs were registered after 12.515 patient-year follow-up. These were assumed to be typical etiologies seen in the general population and unrelated to the congenital lesion [59].

A study conducted on patients with Eisenmenger syndrome, with a median follow-up of 7 years, found that several factors such as AF, QRS duration of ≥120 ms, complete heart block, right bundle branch block (RBBB), impaired biventricular function, and high-burden right ventricular pacing can predict SCD. However, the use of advanced pulmonary hypertension therapies can offer significant protection against SCD, cardiac death, and all-cause mortality [60].

Furthermore, a mutation in the NKX2-5 gene is often seen in ASD patients with conduction disturbances and a higher risk of SCD [61].

The implantable cardioverter-defibrillator (ICD) plays a crucial role in preventing SCD. The recommendations for the ICD in secondary prevention are well established in the current guidelines. However, any stratification scheme for primary prevention ICDs should carefully consider the potential risks as well as the anticipated benefits. It is worth noting that many events leading to SCD are not due to shockable rhythms and hence will not respond to ICD therapy [58]. Moreover, in primary prevention, it is important to consider that ICD-related complications are substantially higher in the CHD population, and this should also be taken into account when discussing the balance between benefits and risks [58]. For individuals who have vascular access limitations, a history of device infections, or a Fontan circulation, the subcutaneous ICD presents itself as an alternative to transvenous systems [58].

## 5. Management of Atrial Arrhythmias Associated with Atrial Septal Closure Devices

### 5.1. Antiarrhythmic Agents and Cardioversion 

Pharmacological therapy as well as cardioversion target rhythm control in patients with atrial flutter/fibrillation following device closure. Initially, anti-arrhythmic agents such as metoprolol, flecainide, and amiodarone can be used, as well as anticoagulation in these patients regardless of the time passed since device closure [62]. When pharmacotherapy fails, electrical cardioversion can be safely undertaken after the process of endothelialization is complete. In small ASOs, this process takes 8 to 12 weeks, whereas bigger devices may require more time, up to 6 months [62,63]. Cardioversion before the completion of device endothelialization may predispose to device displacement and embolization as it is more prone to developing thrombi [62,64].

### 5.2. Interventional Treatment 

The majority of the atrial arrhythmia circuits involve the left atrium, which is less accessible after device closure [22,65]. Atrial arrhythmias (either atrial fibrillation or atrial flutter) are a progressive disease leading to the worsening of left atrial remodeling; hence, aggressive treatment in patients with device closure is essential. Prolonged antiarrhythmics exposure increases the risk of drug toxicity; thus, catheter ablation should be taken into consideration [65]. Patients with device closure are perceived as high risk due to potential dislodgement, while also posing technical challenges for transseptal puncture [65].

#### 5.2.1. Left-Sided Arrhythmia

##### Preprocedural Planning

The ASO is an obstacle to transseptal puncture [9,65]. Access to the left atrium from the right atrium can be gained through the native septum—if there is a portion not covered by the device, or through the device [9,66]. The decision as to where to undertake the transseptal puncture requires the evaluation of structures such as the septum, right atrium, left atrium, aortic root, and the device, as well as their anatomic relationships [66]. To establish the best approach for transseptal puncture site selection, preprocedural planning should include transesophageal or intracardiac echocardiography or/and cardiac computed tomography [9,65,67,68]. 

The timing of the ablation should be carefully calculated. The ASO is completely neo-endothelialized and fibrously incorporated three months after the procedure; thus, access through the device is not likely to cause damage or deformity to the closure device. It has been implied in different studies that at least 3 to 6 months should be allowed after device closure before catheter ablation [66,69].

##### Transseptal Puncture

Transseptal puncture provides access to the left atrium, and it can be performed through the native septum or the device (Table 3).

##### Through the Native Septum

Intraprocedural transesophageal echocardiogram plays a crucial role in describing the septal anatomy and device relation with the septum, helping to identify a suitable puncture site which is most often infero-posterior to the device [9,69]. This is possible for small ASOs that do not completely cover the septum, usually < 26 mm [9], but there have been cases described where native septum puncture was possible even for devices between 26 and 28 mm [66]. The use of radiofrequency energy-assisted septal perforation can be considered, but it may damage the device if used nearby [9]. A case of double transseptal puncture was described through the native septum of a patient with two overlapping closure devices with no peri-procedural complications [68].

##### Through the ASO

For oversized devices that occupy the whole septum and that are usually larger than 26–28 mm, septal anatomy alteration, or, after a failed attempt on the native septum, direct puncture of the device can be performed [9,65,66,69]. This can be achieved using various strategies to overcome the resistance of the device, which include using multiple dilators or sequential dilatations with a non-compliant balloon under pressure (generally angioplasty balloons) [69,70,71,72]. Another technique used is repetitive needle reshaping and adjustment of the puncture angle and position for the puncture to be nearly perpendicular to the plane of the device, allowing in many cases the direct crossing of the device, without extra dilation [66].

##### Mapping and Ablation

After the transseptal puncture, either through the native septum or through the ASO, standard left atrium mapping and ablation techniques should be used. There is no evidence of any significant impact of the device on the electro-anatomical map except for the septal areas covered by the device [9,66].

##### Follow-Up

The reported success rates of catheter ablation are similar to those achieved in patients without atrial septal closure devices [9]. While fear of post-procedural residual shunt, device displacement, and embolization exists, it has been proven unfounded as no such events have been reported. Direct puncture of the device was safe, despite prolonging the procedural time [9,65,69,70,71,73]. In one study, medical therapy after ablation consisted of anticoagulation and antiarrhythmic drug therapy for three months. After this period, medical treatment varied according to stroke risk for anticoagulants and to the treating physician’s clinical decision for antiarrhythmic drugs [66]. Follow-up may include repeated transthoracic echocardiographies, 12-lead Holter monitoring, as well as visits to the physician [66].

#### 5.2.2. Right-Sided Arrhythmia

Right-sided atrial arrhythmias, such as CTI and non-CTI dependent macroreentry arrhythmias and focal atrial tachycardias may also occur in patients with device closure, and standard management techniques can be employed [9].

## 6. Conclusions

- Atrial arrhythmias are common in ASD patients, both before and after ASD closure.
- Clinical, echocardiography, electrocardiogram, and device-related predictors may be used to assess the risk of atrial arrhythmias after ASD closure.
- Device closure of the ASD alone in patients with persistent atrial arrhythmia is not likely to restore sinus rhythm, regardless of the degree of reverse remodeling. This finding suggests that the underlying mechanisms in these patients are complex and at least in part independent of the structural remodeling secondary to hemodynamic overload.
- When required for the interventional treatment of left atrial arrhythmias, transseptal puncture can be performed safely in these patients, either through the remaining native septum or through the ASO.

## Figures and Tables

**Table 1:** Risk factors associated with arrhythmias after percutaneous ASD closure.

Clinical and demographic parameters	Age ≥ 40 yrs [20,21,22] Male gender [23] Metabolic syndrome [24] Thyroid dysfunction [24] Atrial arrhythmia before closure [23,24] Atrial arrhythmia ≤ 1 month after closure [23] BNP levels ≥ 40 pg/mL [19]
Echocardiography parameters	Larger ASD defect [24,25] Multiple ASDs [24] LA and RA dysfunction/remodeling [9,26,27] Mitral valve disease [24] SPAP ≥ 25 mmHg [15,23] Impaired systolic myocardial velocity of RV [15]
Electrocardiography parameters	Increased P-wave duration/dispersion [9,15,28,29] Crochetage sign [30] QT interval, QTc duration and dispersion [29,31] Tp-e interval, Tp-e/QT ratio [31]
Parameters related to the procedure or the device	Larger device size [17,24] Fenestrated occluder [24] ASO/ASL ratio > 0.576 [32] Longer fluoroscopy time [17]

ASD—atrial septal defect; BNP—brain natriuretic peptide; LA—left atrium; RA—right atrium; SPAP—systolic pulmonary arterial pressure; RV—right ventricle; Tp-e—T wave to peak-end interval;; QTc – corrected QT interval; ASO—atrial septal occluder; ASL—atrial septal length.

**Table 2:** Summary of the main studies that analyzed the prevalence of atrial arrhythmias pre- and post-percutaneous ASD closure.

Author (Year)	Study Design	No of Patients Included	Mean Age at the Time of ASD Closure	Target Age That Predicted AA Post-Closure	Follow-Up Period	No of Patients with AA Pre-Closure (%)	No of Patients with AA Post-Closure (%)	Prior CA
De Lezo et al. (2002) [39]	Retrospective	29	56 ± 14 yrs	>40 yrs	21 ± 14 months	12 (41.4)	7 (24.1)	No
Silversides et al. (2004) [40]	Unspecified	132	44 ± 16 yrs	>55 yrs	17 months	20 (15.1)	11 (8.3)	No
Swan et al. (2006) [41]	Retrospective	184	47.1 ± 16.7 yrs	-	6 weeks	10 (5.4)	10 (5.4)	No
Silversides et al. (2008) [36]	Prospective	200	50 ± 17 yrs	>40 yrs	1.9 ± 0.9 yrs	34 (17)	23 (11.5)	No
Balint et al. (2008) [42]	Retrospective	54	59 ± 15 yrs	-	31 ± 15 months	12 (22.2)	9 (16.6)	No
Spies et al. (2008) [18]	Retrospective	240	47 ± 17 yrs	-	20 months	53 (22)	68 (28.3)	No
Giardini et al. (2009) [43]	Retrospective	134	38 ± 16 yrs	-	4.8 ± 2.7 yrs	13 (9.7)	5 (3.7)	No
Mahadevan et al. (2009) [44]	Retrospective	36	46 ± 15 yrs	-	>1 yrs	10 (27.7)	0	No
Altindag et al. (2010) [45]	Retrospective	47	58 ± 13 yrs	-	15 ± 15 months	19 (40.4)	17 (36.1)	No
Humenberger et al. (2011) [46]	Unspecified	238	49 ± 17.4 yrs	-	2.3 ± 1.6 yrs	48 (20.1)	38 (15.9)	No
Vijarnsorn et al. (2012) [47]	Retrospective	353	36.0 yr		1 yr	5 (1.4)	10 (2.8)	No
Van de Bruaene et al. (2013) [23]	Retrospective	131	57.6 yrs	-	40.1 months	16 (12.2)	39 (29.7)	No
Woo et al. (2013) [48]	Retrospective	23	66.7 ± 5.25 yrs	-	21.6 ± 18.5 months	6 (26.0)	2 (8.7)	Yes, just in 1 case
Nyboe et al. (2013) [49]	Retrospective	111	44.2 ± 17.7 yrs	-	3 months	28 (25.2)	21 (18.9)	No
Komar et al. (2014) [17]	Prospective	235	44.6 ± 14.4 yrs		1 yr	58 (24.6)	58 (24.6)	No
Thilen et al. (2016) [50]	Retrospective	148	72.2 ± 4.9 yrs	-	>5 yrs	85 (57.4)	84 (56.7)	No
Wang et al. (2017) [51]	Retrospective	179	53 yrs	-	3–12 months	31 (17.3)	19 (10.6)	Yes, just in 3 cases
Chiu et al. (2017) [24]	Retrospective	517	41.5 ± 14.5 yrs	-	5–64 months	40 (7.7)	27 (5.2)	Yes, just in 3 cases
Duong et al. (2017) [22]	Retrospective	159	57 ± 11 yrs	>40 yrs	1 yr	40 (25.1)	33 (20.7)	Yes, just in 15 cases
Lelakowska et al. (2018) [28]	Prospective	56	49.8 ± 13.3 yrs	-	6 months	18 (32.1)	7 (12.5)	No
Sivakumar et al. (2019) [52]	Retrospective	48	37.24 ± 12.6 yrs		39 months	6 (12.5)	1 (2.0)	No
Fujii et al. (2020) [25]	Prospective	281	58.6 ± 11.3 yrs		2.9 ± 2.1 yrs	34 (12.1)	5 (1.7)	No
Abrahamyan et al. (2021) [53]	Retrospective	1390	47.7 ± 16.3 yrs	40–60 yrs >60 yrs	10.6 yrs	170 (12.2)	182 (13.1)	-
Miura et al. (2021) [19]	Unspecified	238	49 ± 18 yrs		21 ± 14 months	0	13 (5.4)	No
Muroke et al. (2023) [11]	Retrospective	1000	37.9 yrs		5.9 yrs	202 (20.2)	74 (7.4)	-

AA—atrial arrhythmias; ASD—atrial septal defect; CA—catheter ablation.

**Table 3:** Transseptal puncture via native septum versus through the occluder device [9,65,66,69].

	Through the Native Septum	Through the ASO
ASO/ASL ratio	ASO does not completely cover the septum	ASO completely covers the septum
Size of the device	Small ASO (<26 mm)	Large ASO (>26–28 mm)
Time has passed since the device closure	3 to 6 months after device closure	3 to 6 months after device closure
Total procedure time	Shorter	Longer
Puncture site technique	Infero-posterior to the ASO	Assistance with balloon dilation
		“Sequential technique” ^a^
The recurrence of AF	Similar to the ASO approach	Similar to the septum approach

^a^—reshaping the needle and adjusting the puncture angle and position; ASO—atrial septal occlude; ASL—atrial septal length; AF—atrial fibrillation.

## Data Availability

Not applicable.
